# Effect of conditioner and moisture content on flowability of yellow cornmeal

**DOI:** 10.1002/fsn3.1184

**Published:** 2019-09-02

**Authors:** Dipanshu Chinwan, M. Elena Castell‐Perez

**Affiliations:** ^1^ Department of Biological and Agricultural Engineering Texas A&M University College Station Texas; ^2^ Present address: Trans Market LLC 8915 Maislin Drive Tampa Florida 33637

**Keywords:** calcium stearate, flow function, flow index, hygroscopicity, isotherms

## Abstract

Flow characterization of ground materials based on standard physical properties is not always accurate and must be complemented with other properties that help characterize their flowability. The flow properties of yellow cornmeal as a function of moisture content (10.0%, 13.5% 17.0%, and 20.0% wet basis) and concentrations of added conditioner (calcium stearate, a caking agent classified at GRAS, at 0.50%, 0.75%, and 1.00% wt/wt) were measured. The optimum flow behavior characteristics of the cornmeal were achieved at 0.50% wt/wt calcium stearate and 10.0% (w.b.) moisture content based on the flow function test. Overall, the material's flowability decreased with increased moisture content based on very high values for Hausner's ratio, Carr's Index, and angle of repose. The flow index (ff_c_) obtained by the flow function test decreased from 6.47 to 3.82 as moisture increased, indicating increased cohesivity. Calcium stearate increased the flowability of the material at 0.50% wt/wt, beyond which the flowability was not affected (*p* > .05). Samples were very hygroscopic and classified as Class IV. The Hausner's ratio, Carr's Index, and angle of repose showed a strong linear relationship with the flow index with correlation values of 0.91, 0.88, and 0.88, respectively. Isotherms together with physical properties should be used to determine the flow characteristics of granulated materials such as yellow cornmeal to establish the best storage and processing conditions.

## 1 INTRODUCTION

Rat holing, arching, caking, segregation, and flooding are some of the flow problems that are encountered in handling granulated materials. Hence, understanding their properties is very important from an economical and technical point of view (Aulton, 2013; Ghosal, Indira, & Bhattacharya, 2010; Na, Ghadiri, & Wang, 2017). Cornmeal is a coarse flour made from dried corn of particular interest to food engineers and scientists because of its widespread use in manufacturing food products such as tortillas, chips, bread, porridge, and extrudates (Bazua, Guerra, & Sterner, 1979; Carvalho, Takeiti, Onwulata, & Pordesimo, 2010; Ward, Resurreccion, & McWatters, 1998). Most of the literature published on cornmeal is related to the extrusion process (Al‐Muhtaseb, McMinn, & Magee, 2004; Garber, Hsieh, & Huff, 1997; Mathlouthi & Roge, 2003; Onwulata, Mulvaney, & Hsieh, 1994; Zhang & Hoseney, 1998).

Although knowledge of the flow characteristics of cornmeal is essential for selection and design of several unit operations including blending, feeding, compaction, and fluidization, there is still need for more relevant flow property data (Barbosa‐Canovas, Ortega‐Rivas, Juliano, & Yan, 2005; Durney & Meloy, 1986; Molenda, Montross, Horabik, & Ross, 2002; Prescott & Barnum, 2000; Schwedes, 2003; Teunou & Fitzpatrick, 1999; Teunou, Fitzpatrick, & Synnott, 1999). Rheological parameters such as flow behavior index, apparent viscosity, and viscoelasticity of cornmeal dough have been widely researched, but there is a scarcity of literature about the cornmeal as an ingredient itself (Padmanabhan & Bhattacharya, 1993). Therefore, knowledge of the flow behavior of cornmeal will aid in determining whether the material has undergone any significant chemical or physical changes during handling.

Flowability of ground and powdered materials is defined as the ease with which a material will flow under a specified set of conditions (Prescott, Ploof, & Carson, 1999). Hence, a material with good flow characteristics is one that flows consistently without assistance. However, flowability is not an inherent property of a material, so it can never be expressed as a single value or index but rather as a combination of material's physical properties that affect flow (Goodridge, Tuck, & Hague, 2012; Lopes Neto, Meira, & do Nascimento, 2017). Additionally, flowability is affected by particle size distribution, particle shape and density, chemical composition of the particles, moisture, and temperature, among other factors (Aulton & Taylor, 2017; Barbosa‐Canovas, Harte, & Yan, 2012; Fitzpatrick, Barringer, & Iqbal, 2004; Ganesan, Rosentrater, & Muthukumarappan 2008; Opalinski, Chutkowski, & Stasiak, 2012; Sandler & Wilson, 2010; Schulze, 2008; Sokhansanj, Mani, Stumborg, Samson, & Fenton, 2006).

The amount of moisture present in the ground material can drastically change its flow characteristics and storage stability. Moisture isotherms relate to a material's flowability. As the material absorbs moisture from the environment, its flowability decreases because the increased thickness of the material's absorbed liquid layer increases the strength of liquid (capillary) bridges developed between particles. This process continues during storage which can lead to caking and crust formation (Barbosa‐Canovas et al., 2012) or line buildup. Hence, the hygroscopic classification of cornmeal is needed to predict storage stability.

Ground and powdered material flow is also affected by the equipment being used. For instance, wall friction between the particles of the material and the walls of the storage container may impede the flow by providing higher resistance to the particles. While a ground material with good flow properties would be ideal for an application, oftentimes, conditioners are required to increase its flowability. Conditioners or anticaking agents prevent caking and affect flowability as they act as a barrier between the particles, thereby reducing the attractive forces that would cause an inhibition of flow. Conditioners can also reduce the friction between the material particles by acting as lubricants. The following is true in most cases for powders and granulated materials: (a) The finer the powder, the stronger the cohesive forces; and (b) the more inhomogeneous the grain size distribution, the higher the tendency to clump (Hollenbach, Peleg, & Rufner, 1982; Peleg & Mannheim, 1973). These compounds must be effective at low concentrations, and their use is limited to a restricted level of 1.0%–3.0%.

Based on the variability of currently property data, the main goal of this study was to obtain useful engineering data on cornmeal physical and flow properties to better characterize the behavior and predict storage and processing stability of the material.

## 2 MATERIALS AND METHODS

### 2.1 Materials

Quaker^®^ Yellow Corn Meal (The Quaker Oat Company) was purchased at a local market (HEB) and stored at room temperature (25°C) in sealed glass jars until further use. Calcium stearate (Spectrum Chemical Manufacturing Corp.) was used as a flow conditioner (anticaking agent) and stored at room temperature (25°C) until further use.

### 2.2 Experimental design

Experiments for characterization of the flow behavior of the cornmeal consisted of two factors (moisture content and concentration of calcium stearate) at four levels each. Moisture content varied as 10.0%, 13.5%, 17.0%, and 20.0% wet basis, while calcium stearate concentration varied as 0.0%, 0.55%, 0.75% and 1.00% wt/wt, for a total of 48 studies.

### 2.3 Physical and flow properties

#### 2.3.1 Moisture content and sorption isotherms

The moisture content of the cornmeal samples was determined by the oven dry method, modified for use. The modification consisted of drying the sample at 105°C for 24 hr instead of at 130 ± 3°C for 5 hr for complete removal of moisture of the samples without degradation. A calculated weight of RO water from a pipet was added to the samples depending on the moisture content to be achieved, and the moisture throughout the sample was distributed by manual stirring for at least 5 min to achieve uniform moisture distribution (Eaves & Jones, 1972). Next, 4–5 g of sample was placed in round, flat‐bottomed dishes with a tight‐fitting slip‐in cover previously dried at 50°C in an air oven (VWR) for 24 hr, cooled in a desiccator (Vacuum Desiccator, VWR), and weighed with cover using an analytical balance (Sartorius AC 210 S MC 1) soon after attaining 25°C. The cover was leaned against the dish and placed in the air oven at 105°C, and the samples were allowed to dry for 24 hr for complete removal of moisture (AOAC, 1990), transferred to the desiccator with the cover placed to close the dish, and allowed to cool to room temperature. Three repetitions were done for each sample, and moisture content was calculated on a wet and dry basis as,

(1) $$\text{MC}\left(\%w\text{.b}.\right)=\frac{W_{w}}{W_{s}}\times 100$$

(2) $$\text{MC}\left(\%d\text{.b}.\right)=\frac{W_{w}}{W_{d}}\times 100$$

where *W*_w_ is the weight of water in the sample (kg), *W*_s_ is the weight of the sample (kg), and *W*_d_ is the weight of the dry sample (kg).

(Ad)sorption isotherms for the cornmeal at 25°C were constructed using the standard procedure with saturated salt solutions (Labuza, Kaanane, & Chen, 1985). The samples were prepared by drying in a vacuum oven (Lab‐Line Instruments, Inc.) for 6 hr at 70°C. Approximately 5 g of sample was placed in a petri dish and kept in five‐inch desiccators (Thermo Scientific) containing a particular salt solution. The dried samples in the desiccator were placed in an incubator (Curtin Matheson Scientific, Inc.), which was maintained at 25°C. The weight of the samples was determined at 2‐day intervals for about a week until they reached equilibrium (Debnath, Hemavathy, & Bhat, 2002). The moisture content was determined using Equations (1) and (2). Relative humidity values were converted to water activity by multiplying by 100. Three readings for each sample were taken to determine the moisture content. The moisture isotherms can be described as Region I, representing water that exists as a monolayer that is tightly bound to ionic groups such as anions and carboxyl groups (references); Region II representing the multimolecular moisture, which is less firmly bound than the water in Region I, and Region III, in which the moisture exists as free water which is easier to remove (Labuza et al., 1985). The moisture content versus water activity data were fitted to several mathematical models to determine the best predictive equation (Table 1).

**Table 1:** Mathematical models tested for prediction of sorption isotherms at 25°C

Model	Expression	
Modified BET (Aguerre, Suarez, & Viollaz, 1989)	$X=\frac{X_{0}Ca_{w}}{\left[\left(1-a_{w}\right)\left(1-C\ln\left(1-a_{w}\right)\right)\right]}$	(1)
Halsey (Halsey, 1948)	$X={\left[\frac{A}{\left(\ln\left(1/a_{w}\right)\right)}\right]}^{\frac{1}{B}}$	(2)
Smith (Smith, 1947)	$X=A+(B\log\left(1-a_{w}\right))$	(3)
Henderson (Henderson, 1952)	$X={\left[\frac{-\ln(1-a_{w})}{A}\right]}^{\frac{1}{B}}$	(4)
Oswin (Oswin, 1946)	$X=A{\left(\frac{a_{w}}{\left(1-a_{w}\right)}\right)}^{B}$	(5)
Ferro‐Fontan (Fontan, Chirife, Sancho, & Iglesias, 1982)	$X={\left[\frac{\gamma }{\ln(\frac{\alpha }{a_{w}})}\right]}^{\frac{1}{r}}$	(6)
Guggenheim–Anderson–de Boer—GAB (Van den Berg & Bruin, 1981)	$X=\frac{X_{0}CKa_{w}}{\left[\left(1-Ka_{w}\right)\left(1-Ka_{w}+CKa_{w}\right)\right]}$	(7)
	$C=c_{0}\;\exp\left(\frac{\Delta H_{c}}{\mathrm{RT}}\right)$	(8)
	$K=k_{0}\;\exp\left(\frac{\Delta H_{k}}{\mathrm{RT}}\right)$	(9)
Peleg (Peleg, 1993)	$X=K_{1}a_{w}^{n_{1}}+K_{2}a_{w}^{n_{2}}$	(10)

#### 2.3.2 Degree of hygroscopicity

**Figure 1:**
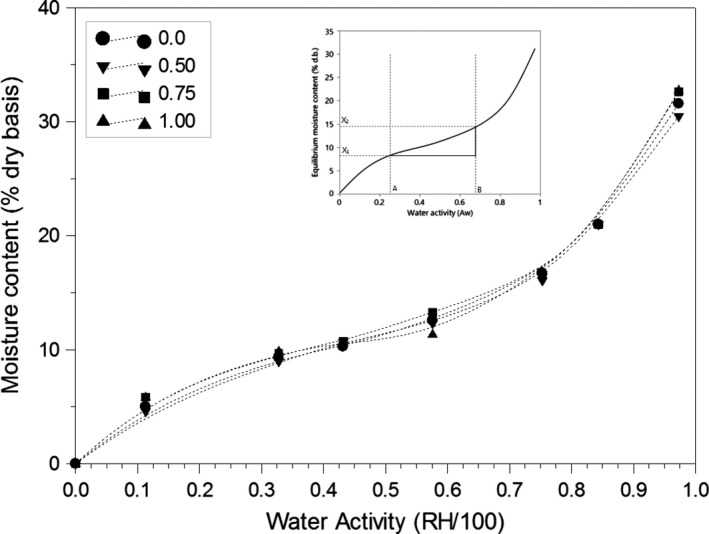
Moisture isotherms and determination of degree of hygroscopicity (g H_2_O/100 g dry sample/*a*_w_) for yellow cornmeal samples at 25°C

A modified version of the method by Callahan et al. (1982) was used in this study so that characterization could be done with minimum amount of sample without mold growth (5 days). The degree of hygroscopicity or the ratio of change in equilibrium moisture content when subjected to a given change in water activity (Equation 3) was obtained from the slope of the moisture isotherm (Figure 1). Materials with high value of degree of hygroscopicity have high water uptake potential and would therefore be classified as highly hygroscopic. The initial and final weights were recorded using an analytical laboratory balance (Sartorius AC 210 S MC 1) and

(3) $$\text{Degree}\;\text{of}\;\text{Hygroscopicity}=\frac{\Delta X}{\Delta a_{w}}$$

where degree of hygroscopicity is in g H_2_O/100 g dry sample/*a*_w,_ and ΔX is the change in the EMC (d.b.) for a given change in water activity (*a*_w_) in the sorption isotherm. The hygroscopicity of the samples was also classified in terms of the amount of moisture absorbed by the sample on achieving equilibrium moisture content (Table 2).

**Table 2:** Classifications of powders in terms of hygroscopicity, Carr's Index (CI), and Hausner's ratio (HR)

Class^a^	Classification
Class I	No moisture increase at R.H. <90%. Increase in M.C. (d.b.) after 1 week above 90% R.H. is <20%
Class II	No moisture increase at R.H. <80%. Increase in M.C. (d.b.) after 1 week above 80% R.H. is <40%
Class III	M.C. does not increase above 5% after storage at R.H. <60%. Increase in M.C. (d.b.) after 1 week above 80% R.H. is <50%
Class IV	M.C. increase may take place at low R.H of 40%–50%. Increase in M.C. (d/b.) after storage for 1 week above 90% R.H. may exceed 30%

Carr's Index^b^	Hausner's ratio	Flow type
CI < 10	HR < 1.11	Excellent flow
11 < CI < 15	1.12 < HR < 1.18	Good flow
16 < CI < 20	1.19 < HR < 1.25	Fair flow
21 < CI < 25	1.26 < HR < 1.34	Passable flow
26 < CI < 31	1.35 < HR < 1.45	Poor flow
32 < CI < 37	1.46 < HR < 1.59	Very poor flow
CI > 38	HR > 1.60	Extremely poor flow

a Hygroscopicity classification for powder based on the relative humidity using desiccators (Callahan et al., 1982).

b Carr's Index and Hausner's ratio to characterize flow type (Shah et al., 2008)

#### 2.3.3 Bulk, tapped, and particle densities and porosity

Bulk density was measured according to the specifications of ISO 3923/1 standard as mentioned by Wong (2002). Approximately 50 g of powder at 25°C was poured in a conical funnel with 1‐inch outlet port, which was blocked at the discharge outlet. A graduated cylinder was placed below the funnel discharge outlet. The powder was filled in the graduated cylinder on the removal of the plug, which was placed to block the outlet of the funnel. Excess powder was removed, and three replicates for bulk density were conducted using the ratio of mass of the powder and the volume of the powder in the cylinder as,

(4) $$\rho_{B}=\frac{W_{s}}{V_{s}}$$

where *W*_s_ is the weight of the sample (kg), and *V*_s_ is the volume occupied by the sample (m^3^).

The tapped density was measured by tapping the graduated cylinder 100 times on a bench so that the powder settles down and no more settling is visible. The tapping process consisted of two taps per second on average. The density was calculated as

(5) $$\rho_{T}=\frac{W_{s}}{V_{t}}$$

where *V*_t_ is the volume occupied after tapping (m^3^).

The particle density was measured using a helium multipycnometer (Quantachrome MVP 4AC232). The procedure involves allowing helium, which is under pressure to flow from the reference cell into a cell containing a sample of material, and the particle density was obtained by taking the ratio of mass of the sample (*W*_s_ in kg) and the average particle volume (*V*_p_, m^3^):

(6) $$\rho_{p}=\frac{W_{s}}{V_{p}}$$

The porosity of the sample was determined using the equation below:

(7) $$\emptyset =\left(\frac{V-V_{p}}{V}\right)\times 100$$

where ∅ is porosity (%), *V* is bulk volume of the bulk (m^3^), and *V*_p_ is particle volume of the bulk (m^3^) from Equation (6). All tests were performed in triplicates.

#### 2.3.4 Angle of repose

The angle of repose, *α*, corresponds to the flow properties of the material and is a direct indication of potential flowability. In general, angles of repose below 30° indicate good flowability, 30°–45° some cohesiveness, 45°–55° true cohesiveness, and >55° sluggish or very high cohesiveness and very limited flowability (Carr, 1965; Geldart, Abdullah, Hassanpour, Nwoke, & Wouters, 2006). Hence, the lower *α*, the easier the material flows. This property is mostly used to design belt conveyors for transport of the materials.

The angle of repose was measured based on ASTM C1444 standard method (ASTM, 2000) by pouring the sample into a funnel, which was held at a fixed height above a flat base as described by Bodhmage (2006). The funnel outlet was kept at a height of 6 cm above the base as per ISO 3435/1. The sample was poured in the funnel at a constant rate manually until the tip of the powder cone touched the funnel nozzle. The diameter of the cone formed was measured at the base in four places to determine the angle of the cone based on the following formula:

(8) $$\propto \;=\;\tan^{-1}\left(\frac{H}{R}\right)$$

where *H* is the height of the cone (m), and *R* is the mean radius of the cone base (m).

The cornmeal was collected on the flat base and the average of the angle between the horizontal and the slope of the sample on both left and right sides taken (Emery, Oliver, Pugsley, Sharma, & Zhou, 2009). This test was repeated three times for each treatment.

#### 2.3.5 Hausner's ratio (HR) and Carr's Index (CI)

The Compressibility Index (Carr's Index) and the Hausner's ratio (HR) are both measures of the material's ability to settle and help to assess the relative importance of interparticulate interactions as they influence flowability (Shah, Tawakkul, & Khan, 2008). When a powder‐like material is free‐flowing, such interactions do not hold huge significance, and the bulk and tapped densities have similar values. On the contrary, when there are poorer flowing materials, there are usually greater interparticulate interactions and the values of bulk and tapped density are very different. These two parameters were calculated as shown below:

(9) $${\text{Hausner}}^{\prime }s\;\text{ratio}\;\left(\text{HR}\right)=\frac{\rho_{T}}{\rho_{B}}$$

(10) $${\text{Carr}}^{\prime }s\;\text{Index}\;\left(\text{CI}\right)=\left[\frac{\rho_{T}-\rho_{B}}{\rho_{T}}\right]\times 100$$

The values for HR and CI given in Table 2 were used to classify the flow behavior of the cornmeal samples.

#### 2.3.6 Particle size distribution

Particle size and distribution affect the flow properties of materials such as cohesiveness, angle of repose, and angle of internal friction. Particle size analysis was carried out using a LS 13 320 multiwave length laser diffraction particle size analyzer (Beckman Coulter Inc.), which uses the theory of Mie scattering, Fraunhofer diffraction, and polarization intensity diffraction scattering (PIDS) (Althaus, 2009). The instrument measures particle size over the range of 0.017–2,000 μm. The scattering pattern is based on the angle at which the light gets scattered, which is characteristic of the particles size. The LS 12 320 uses a 780‐nm laser as light source, which is accompanied by 126 detectors that measure the intensity of light at varying scattering angles. In this study, approximately 10 g of cornmeal powder was placed in a beaker and analyzed. The mean diameter was given by,

(11) $$D\left[4,3\right]=\frac{\sum_{1}^{n}D_{i}^{4}}{\sum_{1}^{n}D_{i}^{3}}$$

#### 2.3.7 Flow function

The flowability of the cornmeal samples was determined with an instantaneous flow function (FF) test using a Brookfield Powder Flow Tester (Brookfield Engineering Laboratories Inc.), which complies with the ASTM D6128 Standard Test Method for Shear Testing of Bulk Solids Using the Jenike Shear Tester test procedure using the annular and Jenike's shear test techniques (ASTM, 2016; Svarovsky, 1987; Xanthakis, Ruud van Ommen, & Ahrné, 2015). The flow function is defined as measure of the internal resistance to flow of a powder, often manifested in its ability to form a blockage in a hopper or feeder (Berry, Bradley, & McGregor, 2015). In other words, it describes the ability of the material to shear on itself at various applied consolidation pressures. The material will flow once the yield stress is overcome by the force acting on it.

The flow function was tested at five uniaxial stresses between 0.3 and 4.8 kPa and three overconsolidation stress levels using a vane lid and running a standard flow function test program. The sample was placed in the trough with volume 263 cm^3^ and tested using the powder flow tester. The sample was first critically consolidated under known normal load and then sheared to fail under four normal stresses, less than the consolidation stress. The shear stress at failure was plotted against normal stress at each consolidation stress to construct a best‐fit yield locus (Crowley, Gazi, Kelly, Huppertz, & O'Mahony, 2014). Five yield loci were created by repeating this process five times. The steady‐state point, consolidation endpoint, and multiple overconsolidation points comprise each yield locus. The flow function curve was plotted between unconfined failure strength (UFS) and the major principal consolidating stress (MPCS) and used to characterize the flow based on the region the curve falls into. The value of unconfined yield strength determines how the flow index, ff_c_, will be affected on applying a particular value of consolidation yield stress. Hence, samples with good flowability will have a lower value of unconfined yield strength obtained from,

(12) $${\text{ff}}_{c}=\sigma_{1}/\sigma_{c}$$

where $\sigma_{1}$ is the consolidation yield stress (kPa), and $\sigma_{c}$ is the unconfined yield strength (kPa). A flow index value >10 indicates excellent flow (free‐flowing material), while a value lower than 1 indicates no flowability at all. In general, easy‐flowing materials have ff_c_ values between 4 and 10 (Fitzpatrick et al., 2004).

#### 2.3.8 Angle of internal friction and angle of wall friction

Other material properties used to characterize flowability are the angle of internal friction and the angle of wall friction. Similar to the angle of repose, a decrease in the angle of internal friction, $\emptyset_{i}$, decreases the resistance between particles, resulting in better flow characteristics. The value of $\emptyset_{i}$ is mostly used for design of bins and hoppers (Emery et al., 2009).

The flow function test was used to calculate $\emptyset_{i}$ using a 23 cm^3^ trough. Unconfined failure strength and major principal consolidating stress were calculated from two specific Mohr circles from each yield locus. The effective angle of internal friction was calculated by measuring the angle between the line, which begins at the origin and is a tangent to the consolidation Mohr circle and the normal stress axis. The angle of internal friction can be measured using the flow function test ff_c_ obtained as described above. The equation representing the angle of internal friction is:

(13) $$\tau =\tan\emptyset_{i}\sigma +C$$

where σ is the shear stress (Pa), $\emptyset_{i}$ is the angle of internal friction, σ is the normal stress (Pa), and C is the cohesion (Pa).

Studies have shown that $\emptyset_{i}$ alone is not the most dominant factor since moist powders exhibit cohesion despite suffering a decrease in the angle of internal friction (Mohsenin, 1970; Peleg & Mannheim, 1973). The angle of wall friction, $\emptyset_{w}$, helps to understand the effect of moisture on the walls of the container holding the sample. A flat wall friction lid was used to measure the friction between the powder particles and the wall using a 263 cm^3^ trough using the Brookfield Powder Flow Tester (Brookfield Engineering Laboratories Inc.). A wall yield locus was constructed for the powder being tested based on the maximum shear stress developed between the bulk powder and the wall material under the three normal stresses before the steady‐state flow occurs. The wall friction angle was calculated by measuring the shear stress required to move the powder continuously across a stainless‐steel surface under three normal stresses, 4.8, 3.2, and 1.6 kPa, applied in order of decreasing normal stress as,

(14) $$\emptyset_{w}=\arctan\;\left(\mu \right)$$

where $\emptyset_{w}$ is the angle of wall friction, and *µ* is the coefficient of wall friction calculated from the slope of the straight line plotted from the origin and intersecting the maximum wall yield locus at a normal stress value of 4.8 kPa.

All tests were carried out with three replications unless otherwise noted. Correlations among the different properties were established using JMP 13 statistical software.

## 3 RESULTS AND DISCUSSION

### 3.1 Moisture sorption and degree of hygroscopicity

All the tested samples showed type II sigmoid isotherms typical of food materials (Figure 1). The curve shows an asymptotic trend as the water activity tends toward unity. At water activity values below 0.7, the rate of moisture absorption, represented by the EMC, is slower compared to the rate of absorption at higher water activity values. Although Peleg's model (Table 1, Equation 11) described the relationship between EMC and Aw of the cornmeal samples very well (*R* ^2^ = .999), the GAB model (Table 1, Equation 8) is a better choice (*R* ^2^ = .999) because it incorporates the monolayer adsorption phenomenon (Table 3). The other models evaluated in this study proved to be less adequate.

**Table 3:** Parameters for the best sorption models for yellow cornmeal at 25°C

Model	Parameters	Calcium stearate (wt/wt)			
		0%	0.50%	0.75%	1.00%
GAB (Equation 8, Table 1)	*X*_0_	6.711	6.860	6.670	6.696
	*C*	34.154	23.122	66.787	63.452
	*K*	0.808	0.796	0.814	0.814
	*SSE*	3.009	3.136	5.234	5.585
	*MSE*	0.601	0.627	1.046	1.117
	*RMSE*	0.775	0.791	1.023	1.056
Peleg (Equation 11, Table 1)	*K*_1_	177.033	123.405	259.649	245.336
	*K*_2_	1.431	1.404	1.383	1.377
	*SSE*	0.524	0.121	0.433	0.756
	*MSE*	0.131	0.030	0.108	0.189
	*RMSE*	0.362	0.174	0.329	0.434

Note

*X*_0_, *K*_1_, and *K*_2_ are parameters for the models (Table 1).

Abbreviation: *SSE*, sum of squared errors of prediction.

Addition of calcium stearate did not (*p* > .05) change the rate of moisture absorption (EMC) of the samples at the test temperature (25°C), and close examination of the slopes of the linear region of the isotherms (Region II; Figure 1 insert) indicates that adding conditioner slightly decreased the degree of hygroscopicity (Equation 3). All the samples are good moisture adsorbers with an average gain of 16 g H_2_O per 100 g dry sample/A_w_. This finding was supported by the Class IV classification materials (Table 2).

### 3.2 Density and porosity

The bulk density of samples without calcium stearate changed (*p* > .05) from 626.14 ± 7.124 to 546.96 ± 5.608 kg/m^3^ when moisture content increased (Table 4). This is an expected finding since the volumetric expansion of the bulk sample is greater than the increase in the mass, resulting in lower bulk density (Kashaninejad, Ahmadi, Daraei, & Chabra, 2008), and the higher the moisture content of the cornmeal material, the greater the need for storage space due to increase in volume of the bulk. Similar to a study on powdered sucrose (Peleg & Mannheim, 1973), the bulk density of cornmeal increased with the addition of calcium stearate, with the effect being more significant (*p* < .05) at higher concentrations of conditioner.

**Table 4:** Physical properties of yellow cornmeal as a function of moisture content and calcium stearate concentration (*T* = 25°C)

Property	MC (% w.b.)	Calcium stearate (wt/wt)			
		0.00	0.50	0.75	1.00
ρ_B_ (kg/m^3^)	10.0	_x_626.14^a^ ± 7.12	_x,y_628.29^a^ ± 4.46	_x,y_633.56^a^ ± 2.01	_y_637.60^a^ ± 4.99
	13.5	_x_610.66^b^ ± 6.05	_x,y_616.70^b^ ± 1.47	_y_621.78^a^ ± 3.75	_y_624.34^b^ ± 7.79
	17.0	_x_573.66^c^ ± 3.33	_y_593.54^c^ ± 5.11	_x_607.91^c^ ± 6.72	_z_614.06^b^ ± 4.62
	20.0	_x_546.96^d^ ± 5.61	_y_581.47^a^ ± 6.07	_z_594.67^d^ ± 1.57	_z_601.95^c^ ± 4.28
ρ_T_ (kg/m^3^)	10.0	_x_695.66^a^ ± 3.76	_x_691.86^a^ ± 5.65	_y_709.73^a^ ± 3.58	_y_716.53^a^ ± 3.51
	13.5	_x_698.73^b^ ± 6.14	_x_699.50^a^ ± 2.19	_y_723.90^a^ ± 9.85	_z_748.86^b^ ± 7.10
	17.0	_x_705.70^a^ ± 9.85	_x_698.46^a^ ± 5.01	_y_745.66^a^ ± 5.06	_z_768.37^c^ ± 7.43
	20.0	_x_708.23^a^ ± 5.82	_y_728.93^a^ ± 5.98	_y_762.10^a^ ± 9.01	_w_792.63^a^ ± 4.08
ρ_p_ (kg/m^3^)	10.0	_y_1,338.8^a^ ± 9.80	_y_1,397.2^a^ ± 7.73	_z_1,419.6^a^ ± 4.14	_y_1,388.5^a^ ± 5.99
	13.5	_x_1,317.3^b^ ± 2.21	_y_1,383.7^b^ ± 1.95	_y_1,390.5^a^ ± 9.63	_z_1,360.4^b^ ± 4.83
	17.0	_x_1,282.1^c^ ± 9.90	_y_1,364.1^b^ ± 3.33	_z_1,379.3^a^ ± 6.96	_w_1,340.8^c^ ± 3.11
	20.0	_x_1,258.8^d^ ± 3.53	_y_1,335.6^b^ ± 10.2	_y_1,325.3^a^ ± 2.41	_z_1,299.2^d^ ± 5.24
ϕ (%)	10.0	_x_53.24^a^ ± 0.38	_y_55.05^a^ ± 0.23	_y_55.41^a^ ± 0.13	_z_54.12^a^ ± 0.20
	13.5	_x_53.69^a^ ± 0.08	_y_55.48^b^ ± 0.06	_y_55.33^a^ ± 0.31	_z_54.13^a^ ± 0.16
	17.0	_x_55.30^b^ ± 0.34	_y_56.52^c^ ± 0.11	_z_55.93^b^ ± 0.22	_w_54.20^a^ ± 0.11
	20.0	_x_56.62^c^ ± 0.12	_x_56.49^c^ ± 0.33	_y_55.18^a^ ± 0.05	_z_53.73^b^ ± 0.19
*ρ_B_ = 666.70 − 5.11MC + 30.93CS; ρ_B_ = 656.26 − 3.69MC + 30.93CS; ρ_T_ = 628.55 − 4.32MC + 54.72CS; ρ_T_ = 637.58 − 3.12MC + 54.72CS; ρ*_p_* = 1,435.54 − 7.87MC + 57.48CS; ρ*_p_* = 1,419.26 − 5.69MC + 57.48CS; ϕ = 53.44 + 0.12MC − 0.41CS; ϕ = 53.69 + 0.08MC − 0.41CS					

Note

^(a–d)^Means within a column, which are not followed by a common superscript letter, are significantly different (*p* < .05). ^(x‐w)^Means within a row, which are not followed by a common subscript letter, are significantly different (*p* < .05).

Abbreviations: ρ_B_, bulk density; ρ_T_, tapped density; ρ_p_, particle density; ϕ, porosity.

* Predictive relationships as a function of moisture content in wet and dry basis, respectively, and CS in percent. Values are mean ± *SD*. Values are means of three replications.

The tapped density of samples without conditioner decreased from 695.66 ± 3.76 kg/m^3^ to 708.23 ± 5.82 kg/m^3^ as moisture content varied. The mean tapped density increased (*p* < .05) at the higher moisture content (17% and 20% wb) and the higher amount of conditioner added. When tapped, the particles come closer together and start to fill the voids between them, resulting in better packing fraction (Bodhmage, 2006). The samples at higher moisture content had liquid bridging between the particles, leading to an increase in bulk volume. This volume decreased when the samples were subjected to tapping causing the bridges to break. Hence, subjecting powdered and granulated materials to vibrations during storage, distribution and handling will result in an increase in tapped density values, which may cause flow problems in silos. Similar trends have been reported for pharmaceutical powders (Emery et al., 2009; Lam et al., 2008).

The particle density of samples also decreased (*p* < .05) with increasing moisture. This decrease can be attributed to the drastic increase in the volume of the cornmeal compared to the mass of the particles. Basically, the volumetric expansion occurs faster than the increase in the mass of the particles. This property also increased with the addition of calcium stearate, with the effect more significant (*p* < .05) at higher concentrations of conditioner. The particles of the conditioner, being smaller in size, adhered to the particles of the cornmeal, which led to an increase in the particle density. After a certain point, the particles of the conditioner were no longer able to adhere to the particles of the cornmeal and segregated out in the bulk sample, leading to a drop in particle density.

The porosity ranged between 53.25% and 56.62%, suggesting irregularities in particle shape since particles with an average porosity of 40% are said to be spheroidal in shape, while a higher porosity value refers to irregular shaped or small particles (Woodcock & Mason, 2012). The samples with 1.00% conditioner and 20% moisture content (w.b.) had closer values of particle density and bulk density, mostly because the particle density was significantly affected (*p* < .05) by the presence of calcium stearate, and high moisture content led to a decrease in particle density.

### 3.3 Hausner's ratio and Carr's index

Higher HR and CI values indicate reduced flowability. Increased moisture content increased (*p* < .05) the Hausner's ratio (HR) and Carr's Index (CI) of samples without conditioner, indicating reduced flowability (Tables 2 and 5). As the particles start to rearrange and fill in the voids, the cornmeal attains higher density, which gives rise to stronger van der Waals forces between the particles, thus increasing the number of contact points (Mani, Rosentrater, & Muthukumarappan, 2006). This force along with an increase in forces due to liquid bridging at higher moisture content results in decreased material flowability (Aulton, 2013; Peleg, 1977).

**Table 5:** Hausner's ratio (HR), Carr's Index (CI), and angle of repose (*α*) of yellow cornmeal as a function of moisture content and calcium stearate concentration (*T* = 25°C)

Property	MC (% w.b.)	Calcium stearate (wt/wt)			
		0.00	0.50	0.75	1.00
HR	10.0	_x_1.11^a^ ± 0.01	_x_1.11^a^ ± 0.02	_x_1.12^a^ ± 0.01	_x_1.13^a^ ± 0.01
	13.5	_x_1.14^b^ ± 0.02	_x,y_1.13^b^ ± 0.01	_y_1.16^b^ ± 0.02	_z_1.20^b^ ± 0.01
	17.0	_x_1.23^c^ ± 0.01	_y_1.17^c^ ± 0.01	_y_1.22^c^ ± 0.01	_z_1.25^c^ ± 0.01
	20.0	_x_1.29^d^ ± 0.02	_y_1.25^d^ ± 0.01	_y,z_1.28^d^ ± 0.01	_z_1.31^d^ ± 0.02
CI	10.0	_x_9.99^a^ ± 0.98	_x,y_9.18^a^ ± 1.25	_x,y_10.73^a^ ± 0.60	_y_11.01^a^ ± 0.65
	13.5	_x_12.59^b^ ± 1.62	_x,y_11.84^b^ ± 0.31	_y_14.09^b^ ± 1.38	_z_16.62^b^ ± 0.86
	17.0	_x_18.70^c^ ± 0.73	_y_15.02^c^ ± 0.25	_y_18.47^c^ ± 0.35	_z_20.08^c^ ± 0.61
	20.0	_x_22.76^d^ ± 0.69	_y_20.23^d^ ± 0.46	_y,z_21.96^d^ ± 0.73	_z_24.05^d^ ± 0.85
α (degree)	10.0	_x_37.00^a^ ± 0.22	_x_37.06^a^ ± 0.30	_x_37.19^a^ ± 0.23	_x_37.26^a^ ± 0.11
	13.5	_x_38.84^b^ ± 0.54	_x_39.20^b^ ± 0.95	_x_38.83^b^ ± 0.32	_z_38.76^b^ ± 0.42
	17.0	_x_39.40^b^ ± 0.21	_x_39.19^b^ ± 0.57	_x_39.77^c^ ± 0.46	_x_39.77^c^ ± 0.33
	20.0	_x_41.53^c^ ± 0.14	_x_41.69^c^ ± 0.42	_x_41.61^d^ ± 0.14	_x_41.69^d^ ± 0.42
*HR = 0.92 + 0.02MC + 0.03CS; HR = 0.96 + 0.01MC + 0.03CS; CI = −3.14 + 1.20MC + 1.87CS; CI = −0.61 + 0.87MC + 1.87CS					

Note

Values are mean ± *SD*. Values are means of 3 replications. ^(a‐d)^Means within a column, which are not followed by a common superscript letter, are significantly different (*p* < .05). ^(x‐w)^Means within a row, which are not followed by a common subscript letter, are significantly different (*p* < .05).

Abbreviations: HR, Hausner's ratio; CI, Carr's Index; α, angle of repose (^o^).

* Predictive relationships between the properties, moisture content, and % calcium stearate at 25°C.

**Figure 2:**
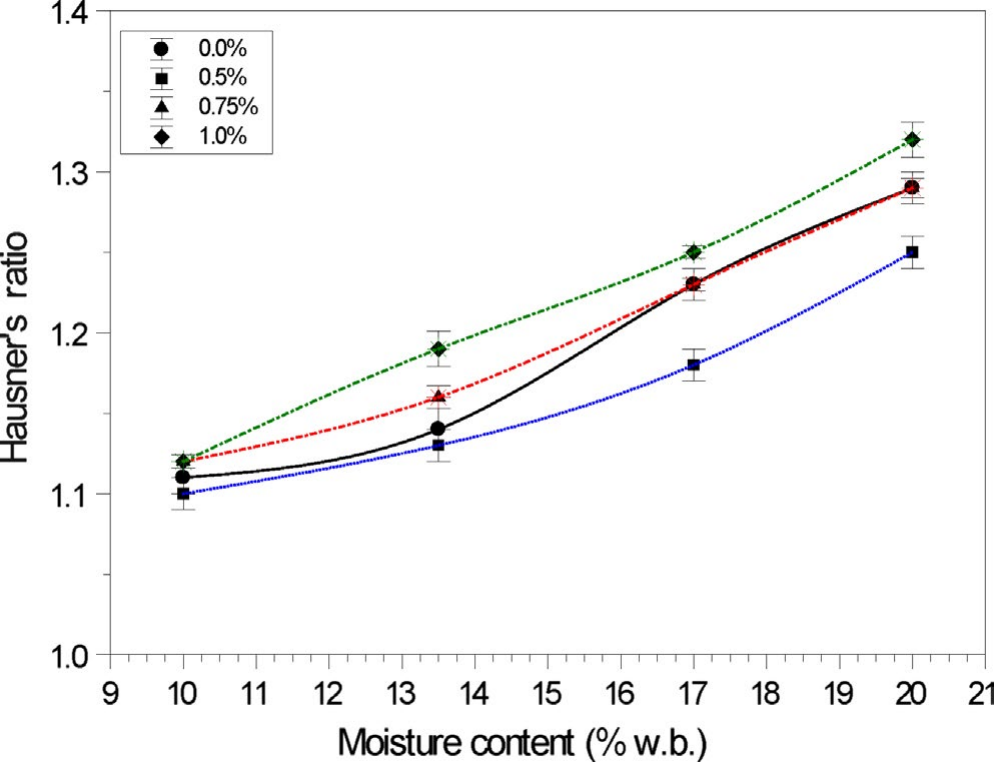
Effect of calcium stearate on the Hausner's ratio of yellow cornmeal at 25°C at varying moisture content

**Figure 3:**
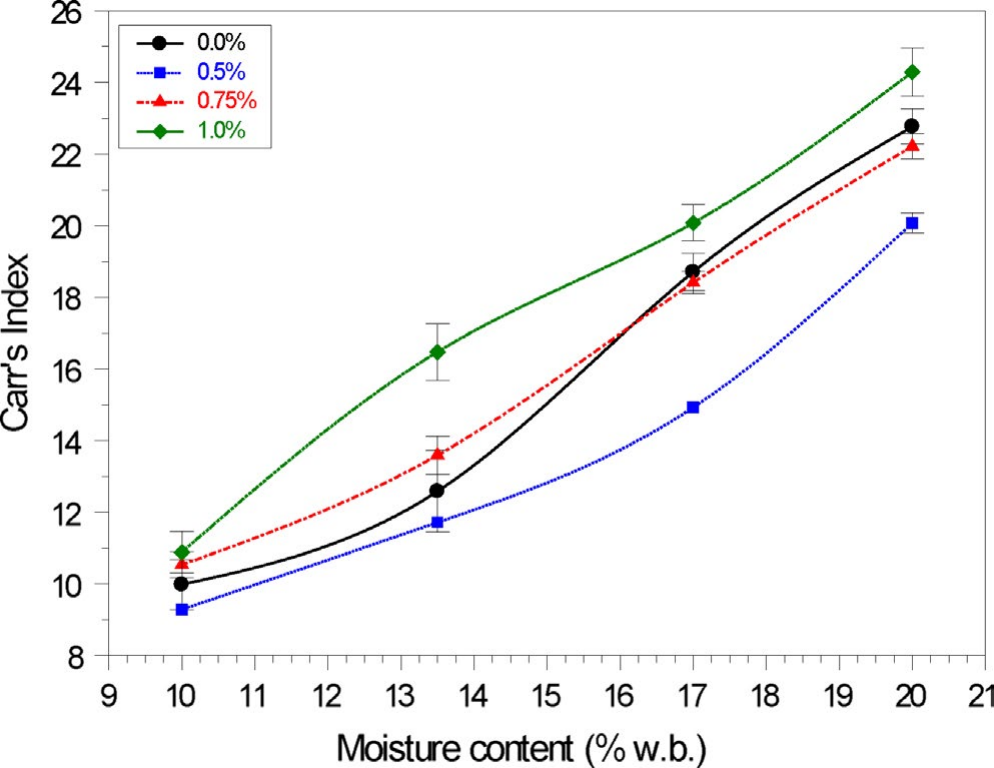
Effect of calcium stearate on the Carr's Index of yellow cornmeal at 25°C at varying moisture content

The impact of adding conditioner to the cornmeal samples is illustrated in the HR values (Figure 2). Similar results were obtained for the Carr's Index (Figure 3). The samples at 10.0% moisture content and 0.50% wt/wt calcium stearate displayed excellent flow properties—lowest mean values for HR and CI. Useful predictive relationships between Hausner's ratio and Carr's Index with moisture content (MC) and concentration of calcium stearate (CS, % w/w) at 25°C were developed (Table 5).

### 3.4 Angle of repose

In general, the more free‐flowing powders tend to possess smaller drained angles of repose. The angle of repose, α, did not change (*p* > .05) when calcium stearate varied at a fixed moisture content (Table 5). This trend is similar to the one observed for Hausner's ratio and Carr's Index. However, at a fixed concentration of calcium stearate, *α* increased (*p* < .05) as moisture content increased due to increased cohesion between the particles, thus making the flowability of the cornmeal sample more difficult. The most unfavorable situation was at 20.0% moisture content because at each concentration of calcium stearate, the value of *α* was highest. It is worth noting that the method used to measure this property yielded very variable results each time, and it is not recommended for use in characterization of cornmeal flow properties.

### 3.5 Particle size and particle size distribution

There is a direct correlation between the size of particles and the flowability of the materials, though moisture content played a more influential role in decreasing the flowability as the particle size increased with increasing moisture content. Table 6 shows that the distribution is mostly narrow for varying moisture contents. Particles with narrow particle size distribution have better flow characteristics than powders with wider particle size distribution (Benkovic & Bauman, 2009). Mean particle size varied from 567.2 to 629.2 µm as moisture content and concentration of calcium stearate varied. Although at fixed concentrations of calcium stearate and varying levels of moisture, the mean particle size increased with increasing moisture content, the change was not significant.

**Table 6:** Mean particle size (µm) and particle size distribution of yellow cornmeal as a function of moisture content and calcium stearate concentration (*T* = 25°C)

MC (% w.b.)	Mean (µm)	*SD* (µm)	Volume < 10% (µm)	Volume < 50% (µm)	Volume < 90% (µm)
Without conditioner					
10.0	592.5	254.2	248.1	605.7	922.2
13.5	590.2	275.5	210.0	597.0	943.4
17.0	600.2	257.6	243.5	614.4	935.4
20.0	610.2	255.4	259.8	623.9	942.0
With 0.50 wt/wt added calcium stearate					
10.0	595.5	242.0	280.7	420.5	909.4
13.5	605.3	240.1	288.7	617.0	914.4
17.0	619.8	239.4	307.3	631.2	929.8
20.0	629.2	242.0	314.3	637.0	941.1
With 0.75 wt/wt added calcium stearate					
10.0	575.5	262.6	202.1	590.8	914.5
13.5	589.1	257.5	232.3	602.9	923.8
17.0	588.0	247.9	251.7	595.9	914.6
20.0	606.0	244.7	307.8	597.2	930.4
With 1.00 wt/wt added calcium stearate					
10.0	567.2	256.4	208.9	578.1	901.6
13.5	581.3	245.0	251.7	590.0	900.9
17.0	585.7	259.9	222.6	597.3	926.3
20.0	592.2	250.0	250.3	600.6	921.5

Note

Values are means of three replications.

Abbreviation: *SD*, standard deviation.

### 3.6 Flow function, angle of internal friction, and angle of wall friction

All the samples can be characterized as easy flowing except for the cornmeal at 20% moisture (ff_c_ value <4) (Table 7), basically due to increased cohesive forces at higher moisture levels. At 20% moisture, the addition of 0.50% conditioner increased the flow index, but then it decreased with increasing amount of conditioner. This finding indicates there is a limit in the ability of calcium stearate particles to fill up the voids and act as a lubricant. Without conditioner, the flow index decreased (*p* < .05) with increasing moisture content. This effect was not very pronounced at higher concentrations of calcium stearate as other factors such as van der Waal forces, mechanical interactions, and the small particle size of calcium stearate overpower its lubricant effect. The sample with 0.50% calcium stearate concentration and 10.0% moisture showed the best flowability with a flow index ff_c_ of 6.70.

**Table 7:** Flow index, angle of internal friction, and angle of wall friction of yellow cornmeal at varying moisture content and calcium stearate concentration (*T* = 25°C)

Property	MC (% w.b.)	Calcium stearate (wt/wt)			
		0.00	0.50	0.75	1.00
ff_c_	10.0	_x,y_6.47^a^ ± 0.19	_x_6.70^a^ ± 0.28	_y_6.14^a^ ± 0.06	_z_5.79^a^ ± 0.13
	13.5	_x_4.70^b^ ± 0.05	_y_5.85^b^ ± 0.30	_y_5.59^b^ ± 0.24	_z_5.15^b^ ± 0.12
	17.0	_x_4.58^b^ ± 0.16	_y_5.00^c^ ± 0.18	_x_4.67^c^ ± 0.071	_z_4.23^c^ ± 0.08
	20.0	_x_3.82^c^ ± 0.05	_y_4.15^d^ ± 0.28	_x,y_3.91^d^ ± 0.14	_x_3.72^d^ ± 0.04
$\emptyset_{i}$(^o^)	10.0	_x_40.37^a^ ± 0.34	_y_39.32^a,b^ ± 0.17	_z_36.86^a^ ± 0.44	_w_38.65^a^ ± 0.07
	13.5	_x_41.26^b^ ± 0.20	_y_39.12^b,c^ ± 0.06	_y_37.42^a,b^ ± 0.46	_z_38.70^a^ ± 0.25
	17.0	_x_39.66^c^ ± 0.24	_y_38.56^c^ ± 0.43	_y_37.96^b,c^ ± 0.28	_z_39.06^b^ ± 0.25
	20.0	_x_37.68^d^ ± 024	_y_39.82^d^ ± 0.42	_z_38.29^c^ ± 0.39	_z_38.97^a,b^ ± 0.02
$\emptyset_{w}$ (^o^)	10.0	_x_12.88^a^ ± 0.08	_y_11.88^a^ ± 0.48	_z_11.30^a^ ± 0.13	_z_11.21^a^ ± 0.18
	13.5	_x_15.41^b^ ± 0.10	_y,z_12.80^b^ ± 040	_z_12.26^b^ ± 0.15	_y_12.93^b^ ± 0.46
	17.0	_x_15.06^c^ ± 0.15	_y_13.50^b^ ± 0.33	_y_13.33^c^ ± 0.28	_x_13.75^c^ ± 0.30
	20.0	_x_17.71^d^ ± 0.15	_y_15.01^c^ ± 0.43	_z_13.58^c^ ± 0.13	_w_14.36^d^ ± 0.12
*ff_c_ = 8.64 − 0.23MC − 0.13CS; ff_c_ = 8.13 − 0.16MC − 0.13CS *t*; $\emptyset_{i}=39.87-0.01\text{MC}-1.46\text{CS};\;\emptyset_{i}=39.86-0.01\text{MC}-1.46\text{CS};\;\emptyset_{i}=10.17+0.31\text{MC}-2.44\text{CS};\;\emptyset_{\text{iw}}=10.84+0.22\text{MC}-2.44\text{CS}$					

Note

^(a–d)^ Means within a column, which are not followed by a common superscript letter, are significantly different (*p* < .05). ^(x‐w)^ Means within a row, which are not followed by a common subscript letter, are significantly different (*p* < .05).

Abbreviations: ffc, flow index; $\emptyset_{i}$, angle of internal friction; $\emptyset_{w}$, angle of wall friction.

* Predictive relationships as a function of moisture content in wet and dry basis, respectively, and CS in percent. Values are Mean ± *SD*. Values are means of three replications.

Without conditioner, the angle of internal friction, $\emptyset_{i}$ decreased (*p* < .05) as moisture content increased (Table 7). Addition of conditioner improved (*p* < .05) the flowability (lower $\emptyset_{i}$ values) of cornmeal, as it decreases particle‐to‐particle friction. The cornmeal with the best flowability is the one at 10.0% moisture content with 0.75% conditioner, while the worst was the cornmeal at 13.5% moisture content without conditioner (highest $\emptyset_{i}$ value).

The angle of wall friction $\emptyset_{w}$ was statistically different (*p* < .05) for increasing moisture content at fixed concentrations of calcium stearate (Table 7). At a fixed moisture content, $\emptyset_{w}$ generally decreased with increased conditioner. At higher moisture contents, the particles strongly adhered to the wall of the instrument, giving a higher $\emptyset_{w}$ value. These findings are supported by the increase in wall friction angle with increased moisture content reported by Davies Grafton and Webster (2009) for biological powder materials and Fitzpatrick et al. (2004) for tea and whey permeate. Useful predictive relationships for moisture content (MC) and concentration of calcium stearate at 25°C were developed (Table 7).

### 3.7 Correlation between physical properties and flow behavior of yellow cornmeal

The physical properties of cornmeal powder do have a major impact on its flowability. Flow properties may be determined using a shear tester, but this procedure is time‐consuming and expensive, whereas physical properties such as bulk and tapped density are easy to measure. Correlations between Hausner's ratio (HR), Carr's Index (CI), particle size, angle of repose (*α*), and angles of internal ($\emptyset_{i}$) and wall friction ($\emptyset_{w}$) with the flow index (ff_c_) were established.

There was a strong positive correlation between HR, CI, and *α*, as the two indices increased with increasing angle. Therefore, either index may be used as a reliable measure of the flow index of cornmeal powder and the relationships can be described as (*R* ^2^ = .90),

(15) $${\text{ff}}_{c}=8.54{\text{HR}}^{-3.07}.$$

(16) $${\text{ff}}_{c}=21.61{\text{CI}}^{-0.54}.$$

Although studies correlate an increase in particle size with increased angle of repose, results from this study indicate that *α* was not significantly affected by particle size. Therefore, it is recommended not to use particle size data to predict the angle of repose of cornmeal samples. Similarly, though the flow index did not correlate well with the internal angle of friction, it showed a strong negative linear relationship with the angle of wall friction; as $\emptyset_{w}$ increased, ff_c_ decreased and the sample did not flow as easily.

## 4 CONCLUSION

Bulk density decreased while tapped density increased significantly (*p* < .05) with increasing moisture content, which affected the flow behavior by resisting flow. In terms of the Hausner's ratio and Carr's Index, the flowability of cornmeal improved with the addition of 0.50% wt/wt of calcium stearate. Beyond this concentration, the effect was not significant, suggesting there is a limit to the conditioning capability of the stearate.

Particle density and porosity were not very significant in predicting the flow behavior. The values of porosity did not follow any specific trend and were difficult to relate to the flow behavior of cornmeal powder. The flowability decreased with increase in moisture content. Particle size increased with increasing moisture content and was the highest for cornmeal powder samples at 0.50% wt/wt calcium stearate. This finding relates well to the flow behavior predicted by Hausner's ratio and Carr's Index. Higher particle size relates to better flowability. Angle of repose decreased with increasing moisture content.

Adding calcium stearate did not affect the sorption isotherms of the tested samples. The samples with 0.50% wt/wt calcium stearate absorbed the lowest moisture in comparison with other samples. Sufficient correlation was not found among all the physical properties, but Hausner's ratio and Carr's Index correlated well with the flow index, providing an easier means to predict the flowability of cornmeal based on the bulk and tapped densities.

It is well known that when hygroscopic powders are exposed to a high relative humidity environment, they will tent do agglomerate and cake, and the presence of anticaking agents does not always help alleviate this issue. Therefore, isotherms together with physical properties should be used to determine the flow characteristics of granulated materials such as cornmeal and determine the best storage and processing conditions.

## CONFLICT OF INTEREST

The authors declare that they do not have any conflict of interest.

## ETHICAL STATEMENT

No human testing and animal testing were conducted in this study.
